# Patterns and correlates of tobacco control behavior among american association of pediatric dentistry members: a cross-sectional national study

**DOI:** 10.1186/1472-6831-7-13

**Published:** 2007-10-11

**Authors:** Stuart A Gansky, Jennifer L Ryan, James A Ellison, Umo Isong, Arthur J Miller, Margaret M Walsh

**Affiliations:** 1 Dept of Preventive & Restorative Dental Sciences, University of California, San Francisco 3333 California Street, Suite 495, San Francisco, CA 94143-1361, USA; 2 2520 Douglas Blvd, Suite 130, Roseville, CA 95661, USA; 3 Department of Orofacial Sciences, University of California, San Francisco, 707 Parnassus Ave San Francisco, CA 94143-0438, USA

## Abstract

**Background:**

To determine the tobacco-related knowledge, attitudes, and practice behaviors among US pediatric dentists.

**Methods:**

A survey was conducted in 1998 among a national, random sample of 1500 American Academy of Pediatric Dentistry members. Chi-square tests and logistic regression with odds ratios (ORs) and 95% confidence intervals assessed factors related to pediatric dentists' tobacco control behaviors.

**Results:**

Response was 65% for the survey. Only 12% of respondents had prior tobacco prevention/cessation training. Of those untrained, 70% were willing to be trained. Less than two-thirds correctly answered any of four tobacco-related knowledge items. Over one-half agreed pediatric dentists should engage in tobacco control behaviors, but identified patient resistance as a barrier. About 24% of respondents reported always/often asking their adolescent patients about tobacco use; 73% reported always/often advising known tobacco users to quit; and 37% of respondents always/often assisting with stopping tobacco use. Feeling prepared to perform tobacco control behaviors (ORs = 1.9–2.8), a more positive attitude score (4 points) from 11 tobacco-related items (ORs = 1.5–1.8), and a higher statewide tobacco use prevalence significantly predicted performance of tobacco control behaviors.

**Conclusion:**

Findings suggest thatraining programs on tobacco use and dependence treatment in the pediatric dental setting may be needed to promote tobacco control behaviors for adolescent patients.

## Background

Tobacco use, the single most preventable cause of premature disease and death in the United States [1], almost always begins during adolescence[2]. Every day in the U.S. more than 3000 youth under age 18 try their first cigarette[2]. It is estimated that half of these youths will become regular addicted smokers, and one-third will succumb to smoking related diseases[2]. Overall, 22% of high school students in the US currently smoke cigarettes and 11% of high school males use smokeless tobacco (i.e., oral snuff or chewing tobacco)[3].

The National Cancer Institute (NCI) recommends all health professionals help patients be tobacco-free by applying the following brief clinical intervention known as the "Five A's Approach" [4]:

▪ Ask all patients about tobacco use;

▪ Advise tobacco users to stop using tobacco and non-tobacco users to remain tobacco-free;

▪ Assess willingness of tobacco users to stop in the next month;

▪ Assist all tobacco users to stop based on their willingness to quit;

▪ Arrange appropriate follow-up.

Many pediatric dentists' patients are adolescents. This frequent contact provides an opportunity to provide a brief tobacco prevention or cessation intervention and reinforcement over an extended period of time enhancing an individual's ability to stop tobacco use [4-6]. The purpose of this study was to evaluate the tobacco control knowledge, attitudes, and behaviors of pediatric dentists and to identify predictors of recommended tobacco intervention behaviors. Although this study was conducted in 1998, a current review of the literature revealed that the most recent published national data on pediatric dentists are from 1994. There is an ongoing need to update the literature to provide benchmarks to monitor trends of compliance with tobacco treatment guidelines over time. This paper presents important new information because it not only reports prevalences of tobacco control behaviors, but also characteristics of providers and their settings related to those behaviors.

## Methods

### Questionnaire development

The University of California San Francisco (UCSF) Institutional Review Board approved this study. To develop and modify the study questionnaire, a convenience-sample discussion group of 7 practicing pediatric dentists was conducted during a 1998 San Francisco Bay Area Pedodontics Study Club meeting. Responses were recorded by the second author (Jennifer L. Ryan) and qualitatively analyzed to inform questionnaire development for pilot testing. Additional questionnaire items were based on a 1994 national survey to assess tobacco control activities among US general dentists[7]. The preliminary questionnaire, cover letter, and pre-paid return envelope were mailed to another convenience sample of 8 other Northern California pediatric dentists to pilot test for clarity, length, and reliability. Results from this pilot test informed final questionnaire development.

### Study overview

The final study questionnaire, cover letter and pre-paid return envelope were mailed in November 1998 to a random sample of 1500 of the 4200 US pediatric dentist members of the American Academy of Pediatric Dentistry (AAPD). All AAPD members were eligible to receive the questionnaire, except retired, student, or foreign members. Two months after the initial mailing, a second questionnaire, modified cover letter, and pre-paid return envelope were mailed to initial survey nonrespondents. Two months later, nonrespondents were randomly sampled (130/551) and telephoned to gather basic demographic information and determine their reasons for not returning the mailed surveys. Consent was implicit in their willingness to complete the telephone interview. Dentists willing to complete a survey were re-mailed the questionnaire. Finally, information on the 1998 state-level tobacco excise tax and the 1997 smoking prevalence for each state were obtained from the US Centers for Disease Control and Prevention website (1999).

### Questionnaire items

The survey defined adolescents as 11–17 years old and tobacco users as smokers (cigarette, pipe, or cigar users) or smokeless tobacco (snuff or chew) users. The questionnaire items assessed demographic factors (age, gender, race/ethnicity) and practice-related demographics (year of dental school graduation, practice type, practice location, weekly work hours). Tobacco-related characteristics of the responding dentist also were assessed, such as tobacco use status, previous tobacco cessation training, willingness to receive training, tobacco control behaviors (identifying/asking, advising, documenting) and perceived barriers to tobacco cessation (11 items using a 3-point Likert scale). Knowledge about tobacco use among adolescents (2 true/false and 2 multiple choice items), attitudes toward tobacco prevention and cessation (9 items using a 5-point Likert scale as per Dolan *et al*. [7] plus 2 additional 5-point Likert scale items) and tobacco-related practice characteristics (office tobacco ban policy and staff member responsible for asking about tobacco use) were also assessed.

### Data analyses

Data entry used built-in error and logic inconsistency checking (Epi Info Version 6, Centers for Disease Control and Prevention, Atlanta, GA). Data analyses used SAS^© ^statistical software (version 9.1.2, SAS Institute, Cary, NC). Assisting tobacco users to stop tobacco use was determined as any of 7 items (discuss quitting strategies, setting a quit date, providing self-help materials, recommending nicotine gum, recommending nicotine patch, referring to a cessation program, or providing follow-up). Nine attitude items (5 point Likert scores) related to tobacco prevention and cessation were summed after reverse scoring 3 items to obtain the Dolan score[7]. In addition, 2 new attitude items asking about encouraging non-users to remain tobacco free and about the importance of tobacco use were added to the 9-item Dolan score to obtain a new 11-item score, the University of California (UC) attitude score. Cronbach's alpha assessed internal consistency of the 11-item UC scale which could range from -22 to 22. Using the American Dental Association's Survey Center geographic definitions[8], nine US divisions were combined into 3 larger regions for analysis.

Categorical measures were assessed with chi-square tests to compare demographics and attitudes about tobacco control between respondents and non-respondents; p ≤ 0.05 was considered statistically significant. Stepwise logistic regression models, with odds ratio (OR) and 95% confidence interval (CI) estimation, were fit separately to identify predictors for each of the 3 tobacco control activities (asking adolescent patients about tobacco use, advising known adolescent tobacco users to quit, and assisting known adolescent users with quitting), with entry and stay criteria of alpha = 0.05. The OR>1 is interpreted as the increased odds of the control activity associated with a change of 1 unit of a numeric explanatory variable or with having a characteristic from a dichotomized explanatory variable. An OR with a 95% CI that does not include one is statistically significant (*i.e*. equivalent to p ≤ 0.05). The following were candidate variables for the logistic regression models: dental school graduation year, geographic location (reference: Atlantic region), state cigarette tax amount (per $0.10 increment), statewide adult smoking prevalence (per 5% increment), health care provider tobacco use, training (previous training and willingness to receive training), intervention self efficacy (feeling very well or well prepared to ask, advise, and assist), gender, office setting (urban or rural; reference: suburban), and the UC attitude scale (for a 4 point increment). In addition, each model was adjusted for 2 barriers associated with non-response: lack of time and lack of tobacco users in the practice (strongly vs. somewhat or not). Collinearlity (*i.e*. condition number) was also assessed.

## Results

### Response rates

Of the 1500 members of the AAPD randomly sampled to receive a survey, 971 returned a survey, for an overall response rate of 65% (952 were returned after the first two mailings plus 19 after the telephone contact). A total of 872 were analyzed after excluding 99 surveys (80 respondent dentists retired or no longer practiced in the US and 19 did not regularly see adolescent patients).

Among the survey's 551 nonrespondents, 130 were randomly chosen to be telephoned. Of these, 14 were ineligible (8 had incorrect or non-working telephone numbers; 6 were no longer AAPD members) and 23 telephone nonrespondents could not be reached (17 unavailable after at least two attempts, 3 relocated and 3 refused). Thus, 93 (i.e. 80% of those eligible) were successfully reached.

Compared to AAPD members returning a survey after telephone contact (phoned respondents), those who did not return a survey (phoned nonrespondents) were significantly more likely to report: lacking time (43% of phoned nonrespondents vs. 16% of phoned respondents), tobacco use was not a problem in their office (10% of phoned nonrespondents vs. 0% of phoned respondents), and lacking interest (23% of phoned nonrespondents vs. 0% of phoned respondents). Nonrespondents and respondents did not differ significantly in thinking tobacco control was an inappropriate topic for pediatric dentists. Phoned nonrespondents and phoned respondents also did not differ significantly in dental school graduation year, age, ethnicity, tobacco use (both current and past), or office tobacco use policy.

### Survey results

#### Description of study sample

Most respondents were White male solo practitioners working 4 or 5 days per week in private, suburban practices. Respondents ranged in age from 28–81 years and graduated from dental school from 1941 to 1998 (Table 1). Eleven percent of respondents were current tobacco users with cigar smoking most frequently reported (Table 2). Almost all respondents banned tobacco use in their practices and about two-thirds reported being personally responsible for asking patients about tobacco use. Only 12% had prior training in tobacco use prevention and cessation. Seventy percent of those without training were willing to be trained. While nearly two-thirds of respondents felt very well or well prepared to ask patients if they used tobacco and to advise users to quit, only 17% felt prepared to assist users to quit.

**Table 1:** Demographic and Practice Characteristics of Responding Pediatric Dentists

**Characteristic**	**%**	**N**
**Age **(years) (N = 858)		
≤ 39	31	264
40–49	32	277
≥ 50	37	317
**Gender **(N = 872)		
Male	75	651
Female	25	221
**Ethnic Group **(N = 868)		
White	86	750
Asian/Pacific Islander	6	51
Latino	3	30
African American	2	17
Other	2	16
Native American	1	4
**Dental School Graduation Year **(N = 862)		
Prior to 1975	35	304
1975–1985	34	294
After 1985	31	264
**Type of Practice **(N = 440)		
Solo	53	233
Group	43	191
Other	4	16
**Setting of Practice **(N = 815)		
Private	89	724
Other*	11	91
**Location of Practice **(N = 865)		
Urban (pop. ≥ 300,000)	44	376
Suburban (> 2,500 but < 300,000)	55	477
Rural (≤ 2,500)	1	12
**Number of Days Worked per Week **(N = 799)		
1 – 3	16	129
4	44	353
5	37	292
6	3	25
**Number of Adolescent^† ^Patients per Day **(N = 850)		
< 10	48	408
10–20	42	353
21–30	7	62
31–40	2	17
> 40	1	10

* Other practice setting included 6% (46) academic, 3% (20) hospital, 1% (10) military, and 1% (10) public health.

^† ^adolescents were defined as 11–17 years old

**Table 2:** Tobacco-Related Characteristics of Responding Pediatric Dentists and Their Practices

**Characteristic**	**%**	**n**
**Current† Tobacco Use‡**		
Any	11	94
Cigarettes	2	15
Cigars	9	77
Pipes	1	6
Smokeless Tobacco	1	5
**Former Tobacco Use‡**		
Any (none current)	15	133
Cigarettes	17	144
Cigars	4	29
Pipes	7	59
Smokeless Tobacco	1	10
**Office Tobacco Policy**		
No tobacco use by patients & parents (N = 862)	99	852
No tobacco use by staff (N = 865)	98	848
Responsible for Asking* (N = 867)		
Pediatric Dentist	67	583
Hygienist	34	293
Dental Assistant	28	241
Health History form	15	126
No one person	13	115
Receptionist	2	13
Other	<1	1
*Do Not Ask*	*17*	*147*

**Prior Training **(N = 864)		
Training in tobacco use prevention or cessation	12	103
**Willingness for First Training **(N = 750)		
Willing to be Trained (among Untrained)	70	527
**Feel Very Well/Well Prepared to**		
Ask about tobacco use (N = 853)	69	587
Advise users to quit (N = 852)	64	546
Assist users with quitting (N = 846)	17	146

*Respondents could check all that applied.

^† ^Current User includes current daily and current occasional users.

^‡ ^Sample sizes: cigarettes N = 844, cigars N = 833, pipes N = 823, smokeless tobacco N = 817

#### Tobacco control knowledge, attitudes, and perceived barriers

Responses to 4 knowledge items about adolescent tobacco use showed relatively low knowledge with only 1 item answered correctly by over half of respondents (Table 3). With regard to attitudes, over half of respondents agreed pediatric dentists should: not use tobacco themselves; encourage abstinence of tobacco use; ask about tobacco use; advise patients who use tobacco to stop; and assist patients wishing to quit. Interestingly, however, over half believed adolescents have a hard time quitting due to addiction and would not quit even with pediatric dentist's advice. Nevertheless, only 20% of respondents thought their time could be better spent doing things other than trying to reduce adolescent patients' tobacco use.

**Table 3:** Pediatric Dentists' Adolescent Tobacco Use Knowledge and Attitudes

**Knowledge Items **(N = 872)	Correct*	
	**%**	**n**
• One in three US adolescents uses tobacco by age 18^†^	60	527
• **90% **of first-time cigarette use occurs before high school graduation^‡^	40	348
• Every day, more than 1000 US adolescents become regular smokers^†^	36	311
• In the last 25 years the number of US adolescents using smokeless Tobacco has **tripled**^‡^	19	168

**Attitude Items**	**Strongly Agree/Agree**	
	**%**	**n**

• The pediatric dentist should set a good example by not using tobacco. (N = 859)	92	793
• It is important for a pediatric dentist to encourage adolescent non-users to remain tobacco free. (N = 859)	78	667
• It is important for a pediatric dentist to ask adolescent patients about tobacco use. (N = 857)	66	565
• Most adolescents will not give up tobacco use even if their pediatric dentist tells them to. (N = 854)	64	548
• It is a pediatric dentist's responsibility to help patients who wish to stop using tobacco to accomplish this. (N = 861)	56	482
• It is a pediatric dentist's responsibility to convince patients who use tobacco to stop. (N = 860)	55	471
• Most adolescent tobacco users have a hard time quitting because they are addicted to nicotine. (N = 856)	54	465
• Pediatric dentists should be more active than they have been in speaking before lay groups about tobacco use. (N = 857)	43	367
• Most adolescent tobacco users can stop if they want to. (N = 851)	37	316
• A pediatric dentist's time can be much better spent doing things other than trying to reduce tobacco use in adolescent patients. (N = 851)	20	173
• Adolescents have enough problems without adding to them by trying to give up tobacco. (N = 859)	4	36

*Missing/"don't know" responses were counted as incorrect.

^†^True/false and ^‡^multiple choice questions (4 items).

Bold indicates correct answer for multiple choice questions.

Internal consistency testing of the Dolan scale had a standardized Cronbach's coefficient alpha of 0.53[7], whereas the UC attitude scale yielded an alpha of 0.67 (moderate internal consistency) – better reliability with the UC scale than that of Dolan *et al*[7].

Seventy-three percent of respondents reported feeling patients' resistance to tobacco cessation services was a major barrier to helping adolescent patients stop tobacco use (Table 4). Moreover, 64% identified feeling they could not effectively help patients quit as a major barrier. Lacking resources, reflected in not knowing where to send patients for counseling and not having materials to distribute, was identified as a major barrier by over half the respondents. Fewer than half the respondents identified lacking time as a barrier and only one-third identified lacking adequate reimbursement.

**Table 4:** Pediatric Dentists' Barriers to Helping Adolescent Patients Stop Tobacco Use* (N = 838)

**Barrier**	%	n
Feel patients are resistant to cessation services	73	608
Don't know where to send patients for counseling	66	550
Don't feel could effectively help patients quit	64	532
Don't have materials to hand out	52	435
Lack of time	48	405
Most adolescent patients do not use tobacco	47	395
Did not occur to me to provide these services	38	322
Don't know what to say	38	320
Lack of adequate reimbursement	34	285
Unsuccessful in providing these services in past	30	251
Don't feel this is appropriate for a pediatric dentist	28	233

*Respondents were asked how much of a barrier each of the following is, or would be, with regard to helping adolescent patients stop tobacco use. Responses included somewhat of a barrier or a strong barrier (excluded not a barrier). Missing responses were combined with 'not a barrier' unless all barriers were missing.

#### Tobacco cessation counseling behaviors

About one-quarter of respondents always or often asked about tobacco use and about one-third assisted users with quitting (Table 5). When aware of tobacco users, however, nearly three-quarters always or often advised them to quit. About one-quarter reported never asking, one-fifth never advising, and one-third never assisting.

**Table 5:** Pediatric Dentists Reported Behaviors Regarding Adolescent Patients' Tobacco Use

	Always/Often*		Never*	
	%	n	%	n
Ask All Patients about Use (N = 857)	24	207	23	193
Advise Users to Quit (N = 856)	73	622	17	17
Assist Users with Quitting (N = 841)	37	313	37	316

* scale: always, often, sometimes, never

#### Factors related to tobacco cessation counseling behaviors

Those respondents who felt prepared to ask, advise, and assist were about 5, 3, and 4 times as likely, respectively, to perform the corresponding task than those who felt unprepared to ask, advise, and assist (Table 6). Respondents who felt it was important to ask adolescent patients about tobacco use, advise adolescent users to quit, and assist users with quitting were about 8, 3, and 4 times more likely, respectively, to perform the corresponding task than those who felt it was unimportant. The 95% CIs indicate statistical significance for all of these associations.

**Table 6:** Relationships of Feeling Preparedand Perceived Importance with Reported Tobacco Control Behaviors

**Behavior**	**Prepared**	**Unprepared**	**OR**^+^	95% CI*
Ask (N = 841)	31	8	4.9	3.1 – 7.9
Advise (N = 839)	81	58	3.1	2.3 – 4.3
Assist (N = 832)	66	31	4.3	2.9 – 6.2

**Behavior**	**Important**	**Unimportant**	**OR**	**95% CI**

Ask (N = 845)	34	6	8.1	4.8 – 13.7
Advise (N = 844)	81	58	3.1	2.2 – 4.2
Assist (N = 838)	46	19	3.6	2.6 – 5.1

*CI = confidence interval

^+^odds ratio

In multivariable logistic regression models (Table 7), adjusting for barriers was usually non-significant (since the 95% CIs include 1.0) – but lacking reported tobacco users in one's practice was significantly related to less asking and assisting (since the 95% CIs exclude 1.0). Feeling prepared to ask, feeling prepared to assist, and the UC attitude score were significantly positively associated with all 3 tobacco cessation counseling behaviors: asking, advising, and assisting. Respondents who felt well or very well prepared to ask or assist were 1.8 to 2.8 times more likely to perform these tobacco cessation counseling behaviors than those who did not feel prepared. A 4 point change in the UC attitude scale (*e.g*. from strongly disagree to strongly agree on 1 of the 11 items or from neutral to agree on 4 items) was associated with being 1.5 to 1.8 times more likely to perform tobacco cessation counseling behavior. Female respondents were 1.7 times more likely to ask about tobacco use than males. Compared to those living in the Atlantic or Central regions, respondents in the Pacific region were 40% less likely to advise. For each 5% increase in statewide smoking prevalence, respondents were 1.5 times more likely to assist users to quit.

**Table 7:** Correlates of Tobacco Cessation Behaviors

**Candidate Variable**	**Ask**		**Advise**		**Assist**	
	**OR**	**95% CI**	**OR**	**95% CI**	**OR**	**95% CI**
Lack of Time	0.7	0.3–1.5	0.9	0.5–1.5	0.7	0.4–1.3
Lack of Users	0.5	0.3–0.9	1.0	0.6–1.5	0.7	0.4-<1.0
Preparation to Ask	2.8	1.4–5.5	1.8	1.1–2.9	1.9	1.2–3.0
Preparation to Advise	1.9	1.1–3.5	1.9	1.3–3.1	2.6	1.7–3.9
UC Attitude Scale*	1.6	1.4–1.9	1.5	1.3–1.8	1.8	1.5–2.1
Gender – Female	1.7	1.1–2.6	---	---	---	---
Region: Pacific vs. Atlantic/Central^†^	---	---	0.6	0.4–0.9	---	---
State Smoking Prevalence^‡^	---	---	---	---	1.5	1.1–2.1

*UC Attitude Scale per 4 point change

^†^Atlantic: Maine, New Hampshire, Vermont, Massachusetts, Connecticut, Rhode Island, New York, Pennsylvania, New Jersey, Delaware, Maryland, District of Columbia, Virginia, West Virginia, North Carolina, South Carolina, Georgia, Florida

Central: Ohio, Indiana, Illinois, Michigan, Wisconsin, Kentucky, Tennessee, Mississippi, Alabama, Minnesota, Iowa, Missouri, North Dakota, South Dakota, Nebraska, Kansas, Louisiana, Arkansas, Texas, Oklahoma

Pacific: Montana, Wyoming, Idaho, Colorado, Utah, Nevada, New Mexico, Arizona, Washington, Oregon, California, Alaska, Hawaii

^‡^State Tobacco Use Prevalence per 5% change

## Discussion

We surveyed members of the AAPD to assess their knowledge, attitudes and behaviors related to tobacco control activities in their dental practices.

Although findings indicate low knowledge levels about general adolescent tobacco use, pediatric dentists held positive attitudes about intervening with their adolescent patients. For example, over half of respondents believed pediatric dentists should encourage, advise, and assist tobacco users to quit using tobacco. In addition, 80% reported that trying to reduce adolescent tobacco use was worth the time. Despite these positive attitudes about intervening with tobacco-using adolescent patients, less than one-quarter of respondents (24%) reported always asking adolescents if they used tobacco. This finding, however, reveals an increase in the behavior of asking patients about tobacco use status compared to the finding of a 1994 survey that reported only 2% of pediatric dentists asked most patients about smoking[9]. The discrepancy might be due to the latter study not differentiating between adolescent and younger patients.

In our study, nearly three-fourths (73%) of respondents reported always or often advising known tobacco users to stop using tobacco, which is consistent with a previously published report on pediatric dentists (80%)[7]. These findings fall just short of the US Department of Health and Human Services *Healthy People 2010 *objective to increase to at least 85% the proportion of dentists who advise cessation[10].

Only 37%, however, reported always or often offering cessation assistance to known tobacco-using patients, similar to other reports[7,9], but significantly less than the 85% *Healthy People 2010 *goal[10]. Perhaps low assisting involvement may have been from feeling unprepared to do so. Interventions are needed to address this void. In fact, over 70% of untrained responding pediatric dentists indicated a desire for training and 55% agreed, "It is a pediatric dentist's responsibility to help patients who wish to stop using tobacco to accomplish this." These findings agree with a 2001 pilot study of 173 pediatric dentists in which 56% felt it was part of pediatric dentists' role to help their adolescent patients stop smoking[6].

In a 1994 study including 586 pediatric dentists as a subgroup, Dolan and colleagues[7] reported, consistent with our findings, that only 12% of pediatric dentists had prior training in tobacco cessation counseling. However, the 2001 pilot study found the percentage increased to 18%, with a third of trained respondents indicating training in the past year[6]. Comparisons of our 1998 data with the 1994 data [7] indicated significant increases in positive attitudes and behaviors related to tobacco control among dentists who have a pediatric interest. For example, feeling very well or well prepared to assist users with quitting significantly increased from 12% in 1994 to 17% in our 1998 study (p < 0.001). In addition, asking patients always or often about smoking and smokeless tobacco use significantly increased from 2% for each in 1994[7] to 8% and 7%, respectively in the current study (p < 0.001). Assisting tobacco users with quitting significantly increased from 9% in the Dolan study[7] to 37% in the current study (both p < 0.001). Comparisons between our study and the 2001 study[6] were not possible due to differences between the two study designs.

Feeling prepared to ask and to advise were significantly predictive of asking, advising, and assisting. Moreover, a moderate association was noted between feeling prepared to ask with asking, and between feeling prepared to advise with assisting. These findings suggest the importance of preparing dentists who see a large number of adolescents on a fairly frequent basis to address tobacco use in their practices. Such training in tobacco cessation could greatly increase the quantity and quality of cessation services to pediatric dental patients.

In addition, the UC attitude score was consistently predictive of all 3 behaviors. This scale could be used to tailor different training program components based on prospective trainee attitude scores since such scores include barriers that could be targeted as educational activities. Female gender was predictive only of asking. Women may have been more likely to ask than men since female health care providers have been reported to be generally more empathetic based on an empathy scale used in a recent study training healthcare providers[11]. Even stratifying by year of graduation to adjust for potential confounding yielded significant gender effects for asking.

Lacking time has consistently been identified as a major barrier to delivering tobacco prevention services[12-14]. Almost half of respondents identified lacking time as a barrier (somewhat of a barrier or a strong barrier). Similarly, nearly half identified feeling most adolescent patients do not use tobacco. One-third of respondents identified lacking adequate reimbursement as a barrier, whereas previous studies reported 39% and 45%[7,9]. The American Dental Association has a tobacco cessation services reimbursement insurance code, but many individual insurance contracts restrict coverage for tobacco prevention and cessation. Dental health policy makers and insurance providers need to address tobacco cessation benefits for patients in oral healthcare settings.

Importantly, 70% of respondents felt patients would resist cessation services. This perception contradicts the literature indicating that most adolescents who smoke want to quit[15,16]. In addition, studies report that patients prefer smoking cessation counseling from a health professional over support groups, self-help, and telephone counseling[17]. A survey of 3,088 dental patients in 53 dental practices revealed that 58.5% believed that dental offices should provide tobacco cessation treatment services. There was equal support among tobacco users and nonusers[17]. Moreover, a survey of patients in independent dental practices and HMO clinics revealed that 40–67% of ST users reported interest in receiving cessation from their dentists. Patients not only expected oral health professionals to advise them on smoking related matters, but welcomed such involvement[18]. Educating pediatric dentists to these patient perceptions may help them overcome their perceived "patient resistance" barrier to helping adolescent patients stop tobacco use. Perceived patient resistance was reported in other studies at 56–94% [14,19].

Respondent dentists in states with higher smoking prevalence were more likely to ask, advise, and assist. Perhaps respondents in these states with high smoking prevalence were more sensitized to the tobacco use problem, and more likely to have received tobacco cessation training, and therefore were more involved in tobacco prevention and cessation activities. In addition, respondents in the Pacific region were less likely to advise about tobacco use compared to those in the Atlantic or Central regions, which may be related to lower state tobacco use prevalence in Pacific region states.

Pediatric dentists can play an important role in preventing initiation or promoting cessation of tobacco use among adolescents to whom they provide care. Adolescents are a unique population. Studies report adolescents consistently rank physical attractiveness, dental concerns, and oral health as greatly important[20,21]. Such findings are highly relevant to pediatric dental practice. They suggest relating smoking to short-term adverse effects associated with attractiveness and oral health may be more relevant and meaningful to an adolescent smoker than relating smoking to long-term health effects such as cardiovascular or lung diseases. Pediatric dentists are well positioned to identify tobacco-related oral health and hygiene problems in the mouths of adolescents who use tobacco. Incorporating this feedback in a brief tobacco cessation intervention in the pediatric dental care setting may encourage adolescents to try to stop smoking. Several studies have demonstrated brief cessation interventions by dental professionals who identify spit (smokeless) tobacco-related lesions in a client's own mouth and who provide brief cessation counseling are effective in helping patients stop their tobacco use[22-25].

Adolescence is the primary time for cigarette smoking initiation with transition from experimentation to some level of dependence[26]. About 65% to 70% of adolescents will try smoking before completing high school, more than one-third will become daily smokers, and almost one quarter will become nicotine dependent[27]. Most adult smokers began smoking by the age of 18[28]. Since adolescent smoking results in increased adult health problems, initiation and maintenance of smoking during adolescence represent a genuine public health concern. The need for effective interventions to prevent the transition from youthful smoking to adult smoking is clearly indicated[28].

Compared with adult smokers, adolescent smokers are more likely to be sporadic or non-daily smokers, and to have more variable smoking patterns on days they do smoke[29]. Nevertheless, many adolescent smokers begin to experience nicotine addiction early in their smoking careers and when smoking only sporadically or occasionally[30-33]. Adolescents may have greater vulnerability to nicotine dependence[34]. Animal studies suggest that processes involved in central nervous system maturation may play critical roles in the development of nicotine dependence[35,36]. Even adolescents who smoke infrequently (*e.g*., only a few cigarettes a month) have a high probability of becoming regular adult smokers. In a large adolescent sample, Chassin *et al*. [37] found that the probability of adult smoking varied by smoking level in adolescence. Findings indicated that adolescents who had smoked more were more likely to be adult smokers, yet 25% of adolescents who had only smoked one or two cigarettes also became adult smokers (defined as smoking in the past week). Given that adolescents are so vulnerable to long term tobacco use, access to them becomes important for early intervention to prevent smoking initiation and to promote smoking cessation. Pediatric dentists are more likely to see teenagers than other health professionals on a regular basis[19].

A limitation of this study was that it was performed in 1998. Publication of the 2000 Clinical Guideline for the Treatment of Tobacco Use and Dependence[4] may have increased training of dentists in the area of tobacco prevention and cessation in dental schools and residency training programs, and in dental practices through dental continuing education. In addition, dental professional associations have actively advertised and supported the dentist's role in tobacco prevention and cessation. A review of scientific sessions sponsored by the AAPD from 2000 to 2006 and of AAPD articles published during the same time period revealed tobacco cessation programs in 2002 and 8 tobacco-related papers (3 on AAPD policy statements in 2000, 2003, and 2006; 3 on the negative health effects of tobacco use on oral health, 2 in 2003 and 1 in 2004; and 2 literature reviews on evidence-based tobacco interventions, 1 in 2000 and 1 in 2006). While these strategies may have increased tobacco-control knowledge among pediatric and general dentists, they appear not to have changed tobacco-control behavior dramatically in general dentists. In the past 15 years, prevalences of general dentists asking patients about tobacco use have not changed radically: ranging from 24% in 1992[38] to 33% in 1994[7] to <60% in 1997[39] to 26% in 2001[40] to 28% in 2005[41]. It is likely that changes in the prevalences of tobacco-control behavior among pediatric dentists over time have paralleled those of general dentists. Nevertheless, no national data on pediatric dentists are available since 1994[7]. Furthermore, factors related to tobacco control activities of pediatric dentists previously have not been reported.

Another caveat of this survey was its reliance on self-reports. Respondents may have overestimated their actual performance when reporting their tobacco cessation counseling behavior. Moreover, because the study sample was drawn from the membership of the AAPD, membership bias may be present in the sample. Dentists likely to join the AAPD may be more likely to perform tobacco cessation behaviors than those who are not members. Additionally, AAPD members include dentists who have pediatric dentistry specialty certificates from accredited programs as well as general dentists interested in treating pediatric patients but who are not certified as pediatric dentistry specialists. Another limitation of our study is that our survey did not distinguish between these two groups. We should therefore be cautious about completely accepting these self-reported values. However, these study results still provide useful information for comparison to other self-reports and for developing and targeting educational programs.

The Public Health Service Guidelines for brief clinical interventions[4] recommend all individuals seeking oral healthcare be asked if they use tobacco. Tobacco users should be advised to quit, assessed for willingness to quit, assisted appropriately based on willingness to quit, and follow-up arranged. Pediatric dentists could not only deter experimental smoking among adolescents by discussing the addiction's dangers, but also could provide referral for treatment for highly dependent smokers. Training programs to enhance knowledge and skills related to the treatment of tobacco use and dependence in pediatric dental settings may be needed and evidence indicates that such programs would be well received by pediatric dentists.

## Conclusion

Respondents who felt prepared to intervene with patients about their tobacco use were much more likely to do so than those who did not feel prepared. Moreover, respondents with training in tobacco cessation counseling were more likely to assist tobacco users with quitting. These and other findings make a strong case for the need to better prepare dentists who treat pediatric patients to address tobacco use in their practices.

## Competing interests

The author(s) declare that they have no competing interests.

## Authors' contributions

SAG designed, analyzed and interpreted the study as well as wrote and edited the manuscript. JLR conceived, designed, performed, and interpreted the study as well as summarized the methods and basic findings in her thesis. JAE performed, analyzed, and interpreted the study as well as edited the manuscript. UI interpreted the study results, expanded the discussion, and edited the manuscript. AJM designed and interpreted the study as well as edited the manuscript. MMW conceived, designed, and interpreted the study as well as wrote and edited the manuscript. All authors have read and approved the final manuscript, except UI who passed away after the original submission.  

## Pre-publication history

The pre-publication history for this paper can be accessed here:


